# Lithium Hydrazinidoborane Ammoniate LiN_2_H_3_BH_3_·0.25NH_3_, a Derivative of Hydrazine Borane

**DOI:** 10.3390/ma10070750

**Published:** 2017-07-05

**Authors:** Salem Ould-Amara, Dominique Granier, Rodica Chiriac, François Toche, Pascal G. Yot, Umit B. Demirci

**Affiliations:** 1 IEM (Institut Europeen des Membranes), UMR5635 (CNRS, ENSCM, UM), Universite de Montpellier, Place Eugene Bataillon, CC047, F-34095 Montpellier, France; salem.ould-amara@iemm.univ-montp2.fr; 2 ICGM (Institut Charles Gerhardt Montpellier), UMR 5253 (CNRS UM ENSCM), Université de Montpellier, CC 15005, Place Eugène Bataillon, F-34095 Montpellier cedex 05, France; dominique.granier@umontpellier.com (D.G.); pascal.yot@umontpellier.fr (P.G.Y.); 3 Univ Lyon, Université Claude Bernard Lyon 1, Laboratoire des Multimatériaux et Interfaces UMR CNRS 5615, LMI, F-69622 Villeurbanne, France; rodica.chiriac@univ-lyon1.fr (R.C.); francois.toche@univ-lyon1.fr (F.T.)

**Keywords:** ammonia carrier, ammoniate, borane, hydrazine borane, hydrazinidoborane, chemical hydrogen storage

## Abstract

Boron- and nitrogen-based materials have shown to be attractive for solid-state chemical hydrogen storage owing to gravimetric hydrogen densities higher than 10 wt% H. Herein, we report a new derivative of hydrazine borane N_2_H_4_BH_3_, namely lithium hydrazinidoborane ammoniate LiN_2_H_3_BH_3_·0.25NH_3_. It is easily obtained in ambient conditions by ball-milling N_2_H_4_BH_3_ and lithium amide LiNH_2_ taken in equimolar amounts. Both compounds react without loss of any H atoms. The molecular and crystallographic structures of our new compound have been confirmed by NMR/FTIR spectroscopy and powder X-ray diffraction. The complexation of the entity LiN_2_H_3_BH_3_ by some NH_3_ has been also established by thermogravimetric and calorimetric analyses. In our conditions, LiN_2_H_3_BH_3_·0.25NH_3_ has been shown to be able to release H_2_ at temperatures lower than the parent N_2_H_4_BH_3_ or the counterpart LiN_2_H_3_BH_3_. It also liberates non-negligible amounts of NH_3_ at temperatures lower than 100 °C. This is actually quite detrimental for chemical H storage, but alternatively LiN_2_H_3_BH_3_·0.25NH_3_ might be seen as a potential NH_3_ carrier.

## 1. Introduction

Hydrogen storage is one of the major obstacles restricting the development of an “economy of hydrogen energy”. Within the past decade, many studies have been done to find new solutions, and particular attention has been paid to chemical H storage [1], and interestingly there has been new interest in old molecules and materials [2]. An example of this is hydrazine borane N_2_H_4_BH_3_. It was discovered in the 1960s and the first applied study presented it as an energetic material [3]. It was then barely investigated until recent years. In 2009, N_2_H_4_BH_3_ was re-discovered owing to its high gravimetric hydrogen density of 15.4 wt%. However, it was considered as being unsuitable for solid-state chemical H storage because of hazardous dehydrogenation properties [4]. Indeed, when heated it releases large amounts of hydrazine N_2_H_4_ together with H_2_, and generates a shock-sensitive solid residue above 300 °C [5]. It is worth mentioning that hydrogen storage via hydrazine borane and any other B–N–H compounds (e.g., ammonia borane NH_3_BH_3_) is irreversible. Upon thermolytic dehydrogenation, they transform into a polymeric residue of complex composition and they cannot be hydrogenated directly under H_2_ pressure. The only way to regenerate the starting materials is chemical recycling [1,2].

Yet, N_2_H_4_BH_3_ is still of interest in the field because it can be modified by reaction with an alkali hydride MH (M^+^ = Li^+^, Na^+^, K^+^) resulting in the formation of an alkali hydrazinidoborane derivative, MN_2_H_3_BH_3_ [6]:

N_2_H_4_BH_3_ (*s*) + MH (*s*) → MN_2_H_3_BH_3_ (*s*) + H_2_ (*g*) (1)

Lithium hydrazinidoborane LiN_2_H_3_BH_3_ is synthesized by ball-milling and depending on the milling conditions, two polymorphs form. The α phase (monoclinic, s.g. (space group) *P2*_1_/*c*), already reported [7], is the high-temperature phase. It can be synthesized directly [7]. Otherwise it can be formed from the β phase (orthorhombic, s.g. *Pbca*) at about 95 °C [8]. The latter phase is the low-temperature phase. Lithium hydrazinidoborane is thermally less stable than the parent N_2_H_4_BH_3_ as a result of the substitution of one H^δ+^ of the N_2_H_4_ moiety by Li^+^ and of the subsequent lengthening and concomitant weakening of the H^δ+^···H^δ−^ interactions (which refer to the so-called dihydrogen bonding). For example, the β phase is able to dehydrogenate from 40 °C and release 7.8 wt% of almost pure H_2_ up to 144 °C. Lithium hydrazinidoborane is thus more suitable for solid-state chemical H storage than the parent N_2_H_4_BH_3_. This is also the case for the sodium derivative NaN_2_H_3_BH_3_ [9]. It starts to liberate H_2_ below 60 °C, and over the range 60–100 °C it loses 6 wt% of almost pure H_2_. The interesting feature with NaN_2_H_3_BH_3_ is that synthesis by ball-milling has to be performed below −30 °C because of the high reactivity of sodium hydride NaH with N_2_H_4_BH_3_. A reaction enthalpy of −27,756.7 J mol^−1^ was determined by Calvet calorimetry at 25 °C [10]. The reaction is highly exothermic, especially when compared to the enthalpy of the reaction between LiH and N_2_H_4_BH_3_ at the same temperature (−62.8 J mol^−1^). In fact, the bigger the alkali cation is, the more exothermic the reactivity of the alkali hydride. The enthalpy of reaction of KH with N_2_H_4_BH_3_ was measured as being −70,247.2 J mol^−1^. Accordingly, the safest way to get potassium hydrazinidoborane KN_2_H_3_BH_3_ is by wet synthesis (i.e., suspension in tetrahydrofuran) in an autoclave reactor [11]. All of these alkali hydrazinidoboranes have shown better and safer dehydrogenation properties than the parent hydrazine borane. To our knowledge, there is no other derivative of N_2_H_4_BH_3_. Attempts in synthesizing the hydrazinidoboranes of magnesium, calcium, and aluminum M(N_2_H_3_BH_3_)*_n_* (M*^n^*^+^ = Mg^2+^, Ca^2+^, Al^3+^) by ball-milling have failed. Composites MH*_n_*-N_2_H_4_BH_3_ have been found to form where the hydrides act as destabilizers of the borane [10].

The substitution of one H^δ+^ of the N_2_H_4_ moiety of N_2_H_4_BH_3_ by M^+^ negatively affects the gravimetric hydrogen density of the as-formed material because of the loss of 1 equivalent of H_2_. For instance, the gravimetric hydrogen density drops from 15.4 wt% for N_2_H_4_BH_3_ to 11.7, 8.9, and 7.2 wt% for LiN_2_H_3_BH_3_, NaN_2_H_3_BH_3_, and KN_2_H_3_BH_3_, respectively. The decrease might be compensated by using an amide MNH_2_ instead of a hydride MH, only if the NH_2_ group of the amide interacts by trapping one H^δ+^ of the N_2_H_4_ moiety of hydrazine borane. Consequently, the formation of a new compound may be expected with a formula resembling MN_2_H_3_BH_3_·*x*NH_3_. Such an approach has shown to be successful for synthesizing metal amidoborane ammoniates like Mg(NH_2_BH_3_)·NH_3_ and Ca(NH_2_BH_3_)·NH_3_ where all of the hydrogen atoms of the precursors were kept in the final material [12,13]. We therefore explored the possibility of synthesizing novel derivatives of N_2_H_4_BH_3_. Lithium amide LiNH_2_ was selected owing to the lightness of Li^+^. Another reason for this choice is that LiH is less reactive than NaH or KH, and thus is safer. Similar behavior was expected for LiNH_2_. Then, by ball-milling N_2_H_4_BH_3_ and LiNH_2_, the formation of lithium hydrazinidoborane ammoniate LiN_2_H_3_BH_3_·*x*NH_3_ was expected; it has a higher gravimetric hydrogen density (13.2 wt% for *x* = 1; 12.1 for *x* = 0.25) than LiN_2_H_3_BH_3_ (11.6 wt%). This is discussed hereafter.

## 2. Results

### 2.1. Molecular Structure

Ball-milling of LiNH_2_ and N_2_H_4_BH_3_ in our conditions resulted in the formation of a pasty solid. More details are given in the section dedicated to the experimental conditions. The pasty aspect is otherwise discussed in the discussion section. Hereafter, the precursors N_2_H_4_BH_3_ and LiNH_2_ will be denoted **1** and **2** respectively, while the ball-milling product will be identified as **3**.

**Figure 1:**
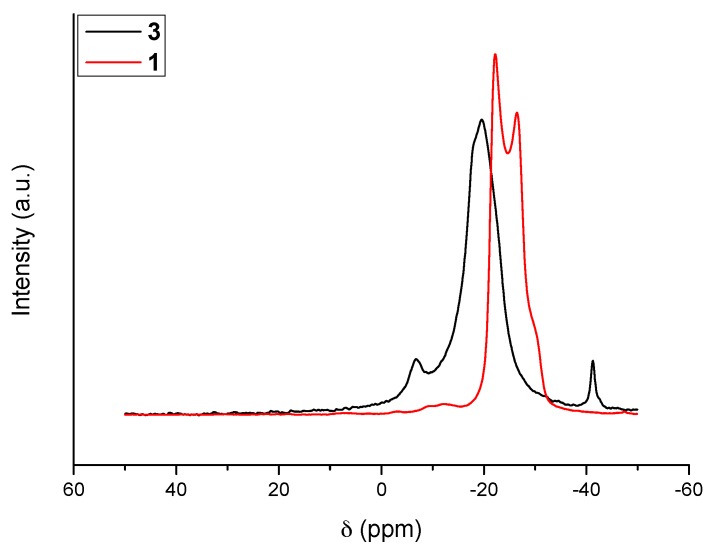
^11^B MAS NMR spectra of **3**. For comparison the spectrum of N_2_H_4_BH_3_ (**1**) is shown.

The ^11^B MAS NMR spectra of **1** and **3** are shown in Figure 1. With the former (i.e., N_2_H_4_BH_3_), the signal is typical of a quadrupolar coupling due to anisotropy around the boron atom because of strong intermolecular H^δ+^···H^δ−^ interactions. In contrast, the new compound **3** shows a resonance of high intensity at δ −19.6 ppm; the signal is otherwise broad. Such features are indicative of isotropy around the boron atom of the N*B*H_3_ environment, change of the environment of the N–H bonds, and lengthening of the intermolecular H^δ+^···H^δ−^ interactions. Similar observations were reported for LiN_2_H_3_BH_3_ as well as for NaN_2_H_3_BH_3_ [6,7,8,9]. There are also two other signals. The first one is centered at δ −41.3 ppm. It is typical of a *B*H_4_ environment [14]. The second one is centered at δ −6.7 ppm. It may be ascribed to N_2_*B*H_2_ and N_3_*B*H environments [15]. The presence of such N_2_*B*H_2_ and N_3_*B*H environments might indicate some decomposition of the starting borane, but the concomitant apparition of the *B*H_4_ environment does not support such an assumption. In the open literature dedicated to B–N–H compounds, the presence of both signals is generally ascribed to the formation of an ionic dimer [16]: for example, with ammonia borane NH_3_BH_3_, a similar ^11^B MAS NMR spectrum can be collected when part of the borane transforms to the ionic dimer diammoniate of diborane of formula [NH_3_BH_2_NH_3_^+^][BH_4_^−^]. We therefore believe that in our conditions a little part of N_2_H_4_BH_3_ dimerized into an ionic intermediate of formula [N_2_H_4_BH_2_N_2_H_4_^+^][BH_4_^−^] (dihydrazinoboronium borohydride or dihydrazinate of diborane):

2N_2_H_4_BH_3_ (*s*) → [N_2_H_4_BH_2_N_2_H_4_^+^][BH_4_^−^] (*s*) (2)

Such a compound shows both of the N_2_*B*H_2_ and *B*H_4_ environments, while the BH_3_ environment comes from the new solid **3**.

**Figure 2:**
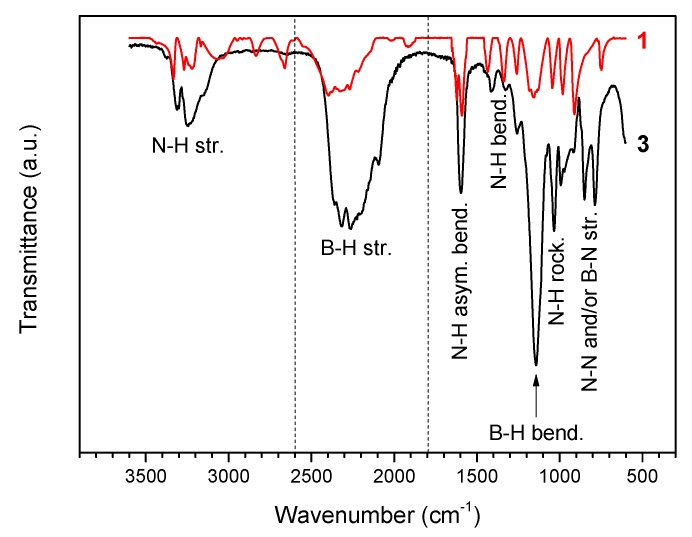
FTIR spectrum of **3** and, for comparison, that of **1** (N_2_H_4_BH_3_). The different vibrational modes are indicated.

The FTIR spectrum of **3** (Figure 2) is different from that of **1**. It displays a less complex fingerprint in the N–H stretching region (3450–2950 cm^−1^) and broadened N–H bending bands (1700–1300 cm^−1^), thereby suggesting that the intermolecular H^δ+^···H^δ−^ interactions are less important and that the H^δ+^···H^δ−^ network is weaker. There are more bands in the B–H stretching region (2600–1800 cm^−1^) owing to interactions of Li^+^ and N of **2** with H^δ−^ of BH_3_ groups. The two small bands at 1913 and 2017 cm^−1^ observed on the spectrum of N_2_H_4_BH_3_ are indicative of strong H^δ+^···H^δ−^ interactions [5]. They cannot be seen for **3**, confirming a weakened H^δ+^···H^δ−^ network. It may be reasonably concluded that **2** has induced electronic modification in **1**. In fact, the FTIR spectrum of **3** resembles that of LiN_2_H_4_BH_3_ reported elsewhere and similar conclusions were made for this derivative [8]. The N–H stretching vibration at 3375 cm^−1^ is in the range of the degenerate stretching N–H mode of ammonia, thereby suggesting weakly bound NH_3_ molecules [17].

The aforementioned spectroscopy results are indicative of the formation of a compound with the speculated molecular formula LiN_2_H_3_BH_3_·*x*NH_3_ (lithium hydrazinidoborane ammoniate).

### 2.2. Crystallography

**Figure 3:**
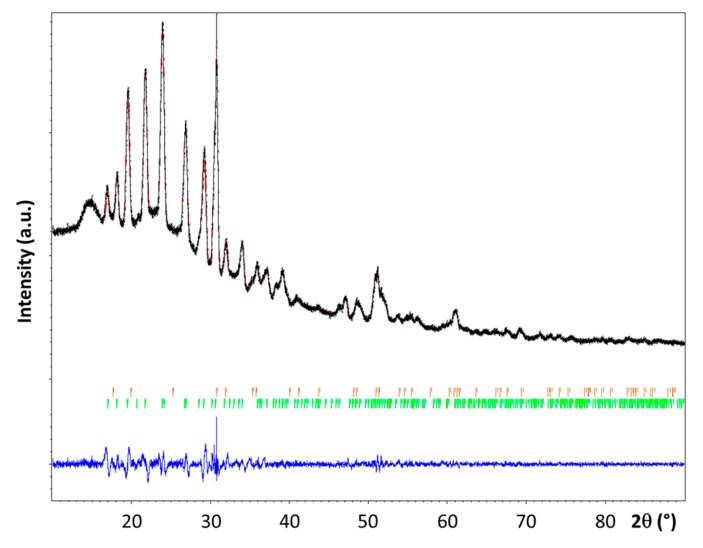
Observed (in black) and calculated (in red) powder X-ray diffraction profiles for the Rietveld refinement of the LiN_2_H_3_BH_3_·*x*NH_3_ phase. The bottom curve (in blue) is the difference plot on the same scale intensity and the tick marks (in green for LiN_2_H_3_BH_3_·*x*NH_3_ and in orange for LiNH_2_) are the calculated angles for the Bragg peaks in 2θ (λ = 1.5418 Å).

The powder XRD pattern recorded for **3** is shown in Figure 3. It was compared to the patterns of the reactants **1** [3,7,18] and **2** [19]. The presence of a new crystalline phase was confirmed, though some residual diffraction peaks coming from **2** have been also detected.

The diffraction peaks of **3** have been successfully indexed as a single phase using DICVOL06 [20] after removing the peaks supposed to be overlapped with the LiNH_2_ diffraction lines. The new phase **3** crystallizes in the monoclinic system, with a space group *P2*_1_/*n* (No. 14) and Z = 4; the unit cell parameters a = 7.649(1) Å, b = 7.502(1) Å, c = 5.973(1) Å, and β = 97.81(1)° were found. The unit cell volume (~340 Å^3^) is larger than the one reported for α–LiN_2_H_3_BH_3_ (328 Å^3^) [7]. The structure factor of **3** and that of the α [7] and β [8] phases of LiN_2_H_3_BH_3_ were compared; they present strong differences. This is indicative of the formation of a new phase. The position of the Li element in the unit cell was located by Direct Methods using EXPO2014 [21]. The crystal structure was then successfully refined by the Rietveld method (Figure 3) using the Jana 2006 program [22]. The structure parameters that were obtained are presented in Table 1 and Table 2. For the refinement the lengths of the N–N and N–B bonds and the N–N–B angle were fixed to 1.50 Å, 1.58 Å, and 113.8° respectively. The refinement was carried out while taking into account the presence of **2**. As a result, the relative weight amounts of **3** (LiN_2_H_3_BH_3_·*x*NH_3_) and **2** (LiNH_2_) were found to be close to be 95.4 and 4.6 wt%, respectively. Nevertheless, the refinement of the position of the *x* molecule of NH_3_ into the crystalline network has not been possible. Hence, considering the difference of volume (14 Å^3^) between the cell volume of **3** and that of α–LiN_2_H_3_BH_3_, it can be assumed the presence of one non-H atom per unit cell. This atom is proposed to be N of NH_3_, suggesting then the molecular formula LiN_2_H_3_BH_3_·0.25NH_3_ for **3**.

### 2.3. Thermal Analyses and Evolving Gas Analyses

**Figure 4:**
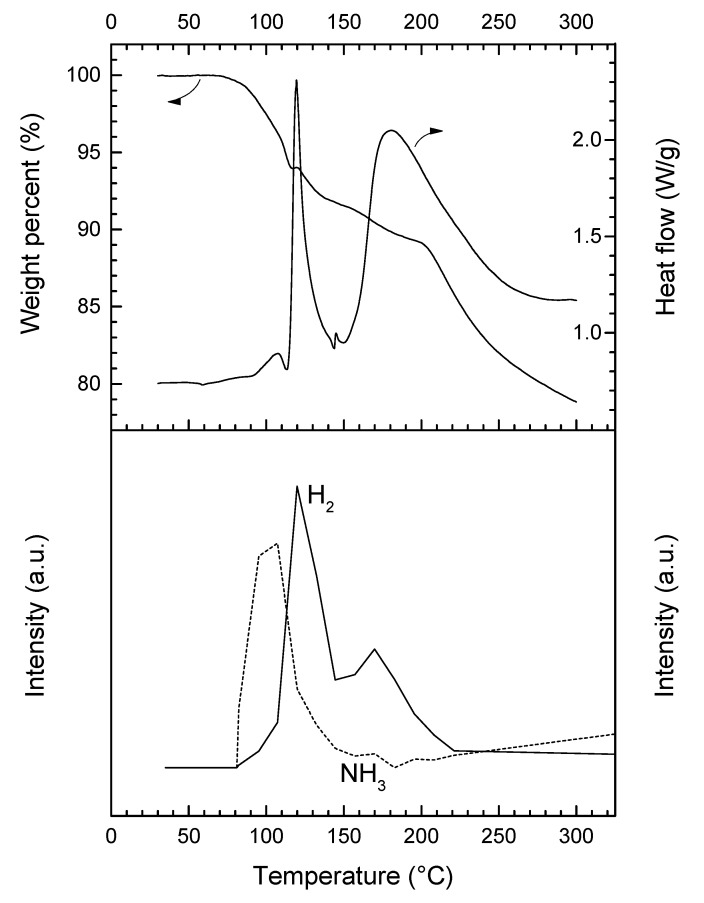
TGA, DSC, and µGC-MS (H_2_ *m*/*z* = 2, NH_3_ *m*/*z* = 17) data for **3** (heating rate of 5 °C min^−1^).

Under heating at 5 °C min^−1^, **3** is stable up to 75 °C (Figure 4). Then, it decomposes according to a complex pathway. The DSC curve is characterized by two major (maximum at 119.4 and 180.2 °C) and two minor exothermic events (107.4 and 145.5 °C). This is consistent with the occurrence of several weight losses observed by TGA. Over 75 °C, **3** starts to liberate NH_3_, which is characterized by the first exothermic event (0.9 kJ mol^−1^) peaking at 107.4 °C. This is consistent with the molecular structure of an adduct like LiN_2_H_3_BH_3_·*x*NH_3_. The first major weight loss (8.3 wt%) that occurs over the temperature range 75–145 °C is associated with the second exothermic event (11.5 kJ mol^−1^) peaking at 119.4 °C. It is mainly due to the dehydrogenation of **3**. There is then an exothermic signal of low heat (0.15 kJ mol^−1^). It is followed by the second major decomposition (12.9 wt% and 50.5 kJ mol^−1^) that takes place over the temperature range 145–300 °C. At 300 °C, the overall weight loss is 21.2 wt% and it cannot be rationalized with the loss of H_2_ only (maximum of 13.2 wt% H in **3** if *x* = 1; 12.1 wt% H if *x* = 0.25). The weight proportion of NH_3_ in the adduct LiN_2_H_3_BH_3_·*x*NH_3_ is theoretically of 24.7 wt% NH_3_ if *x* = 1, and 7.6 wt% NH_3_ if *x* = 0.25. Hence, **3** releases H_2_ and significant amounts of NH_3_.

In our conditions, **3** releases most of the NH_3_ during the first decomposition step. Similar trends were reported for lithium amidoborane ammoniate Li(NH_2_BH_3_)·NH_3_, for which the evolution of NH_3_ peaks at 52 °C while that of H_2_ shows the maximum at around 100 °C [23]. Similar observations were reported for calcium amidoborane ammoniate Ca(NH_2_BH_3_)·NH_3_, making the authors suggest that the adducted NH_3_ is weakly bound to the cation Ca^2+^ [13].

**Figure 5:**
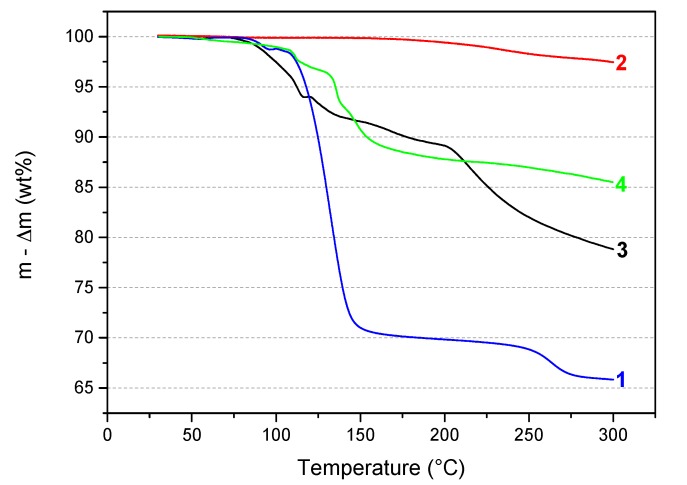
Superimposition of the TGA curves of **1** (N_2_H_4_BH_3_; from ref. [5]), **2** (LiNH_2_), **3** (LiN_2_H_3_BH_3_·*x*NH_3_), and **4** (LiN_2_H_3_BH_3_; from ref. [8]).

In our laboratory, TGA has been used as an efficient screening tool to evaluate the potential of any new B–N–H compound as well as to compare several of them. Accordingly, the thermal behavior of the sample **3** was first compared to that of the reactants **1** and **2**. The TGA curves are shown in Figure 5. In comparison, **2** is quite stable under heating at 5 °C min^−1^. The weight loss of about 0.6 wt% is negligible before 200 °C and increases to 2.5 wt% up to 300 °C. With respect to **1**, it has a much different TGA profile. Therefore, **3** is a compound that is different from the precursors. As mentioned in the previous paragraphs, **3** is somehow comparable to the ammonia-free derivative LiN_2_H_3_BH_3_ (denoted **4** in Figure 5). So, the thermal behavior of **3** was also compared to that of **4**. It is worth mentioning that **4** is known to dehydrogenate according to a complex mechanism not releasing unwanted by-products like NH_3_ [7,8]. The thermal behaviors of these two compounds are different. **3** starts its decomposition at a lower temperature than **4** and shows a higher weight loss due to the release of NH_3_ molecules from both the complexation and the decomposition phenomena. In other words, the presence of NH_3_ in **3** leads to a thermolytic behavior which is different from that observed with **4**.

## 3. Discussion and Concluding Remarks

In good agreement with our primary objective, a new hydrogen-rich B–N–H compound has been successfully synthesized by solid-state reaction (mechanosynthesis/ball-milling) of N_2_H_4_BH_3_ and LiNH_2_. The molecular analyses suggest a compound with the formula LiN_2_H_3_BH_3_·*x*NH_3_. This is a new type of coordination compound consisting of a metal cation Li^+^, a [N_2_H_3_BH_3_]^−^ anionic unit, and an *x*NH_3_ ligand [24]. The crystallographic analysis allowed quantifying the *x* value thanks to the difference in the cell volumes of LiN_2_H_3_BH_3_·*x*NH_3_ and another well-described derivative—LiN_2_H_3_BH_3_. Indeed, *x* was found to be equal to 0.25 suggesting then the following reaction:

LiNH_2_ (*s*) + N_2_H_4_BH_3_ (*s*) → LiN_2_H_3_BH_3_·0.25NH_3_ (*s*) + 0.75NH_3_ (*g*) (3)

Note that we also worked on a mixture of LiNH_2_ and N_2_H_4_BH_3_ (results not reported) where the mole ratio was lower than 1. We indeed considered 0.75 mole of LiNH_2_ and 1 mole of N_2_H_4_BH_3_. Like for LiN_2_H_3_BH_3_·0.25NH_3_, a paste-like solid was obtained. The as-obtained product showed NMR and FTIR spectra that were comparable to those of LiN_2_H_3_BH_3_·0.25NH_3_. With respect to the powder XRD pattern, the diffraction peaks were comparable to those observed for LiN_2_H_3_BH_3_·0.25NH_3_, but with additional peaks belonging to the excess of N_2_H_4_BH_3_. The TGA results were also comparable. In other words, the results and the observations were consistent with the occurrence of the reaction shown by Equation (3).

The reaction of LiNH_2_ and N_2_H_4_BH_3_ in our conditions can be interpreted as follows. The Lewis base NH_2_ of LiNH_2_ would favorably react with the acidic hydrogen H^δ+^ of the N_2_H_4_ moiety of the borane resulting in NH_3_. In parallel, the Li^+^ cation would combine with the H^δ+^-deficient N_2_H_3_^−^ entity towards the formation of the entity LiN_2_H_3_BH_3_. It seems that a quarter of the as-formed NH_3_ complexes LiN_2_H_3_BH_3_ leading to the formation of a LiN_2_H_3_BH_3_·0.25NH_3_ like-compound. The rest of the NH_3_ (the 0.75 equivalent) can be evacuated under vacuum at ambient conditions.

Ammonia is known to have good affinity with the B–N–H compounds. A first example is ammonia borane. Exposed to an atmosphere of NH_3_, NH_3_BH_3_ is able to complex up to six molecules of NH_3_, resulting in the formation of a pasty solid [25]. Furthermore, NH_3_ is an excellent solvent of NH_3_BH_3_; the solubility is 259.7 g of NH_3_BH_3_ in 100 g of NH_3_ and the solvated borane shows good stability [26]. A second example is an alkali derivative of NH_3_BH_3_. It was observed that exposure of LiNH_2_BH_3_ to NH_3_ produces a sticky liquid containing a 1:1 molar ratio of LiNH_2_BH_3_ to NH_3_ [23]. Keeping in mind these reported observations, we did a simple experiment. We synthesized LiN_2_H_4_BH_3_ as reported in our previous report [8] and exposed it to a stream of NH_3_. Like for the aforementioned LiNH_2_BH_3_ [23], our sample changed to a sticky liquid/pasty material. Hence, we may conclude that the “excess” of 0.75 mole of NH_3_ (Equation (3)) explains the paste-like state of **3** right after ball-milling.

**Figure 6:**
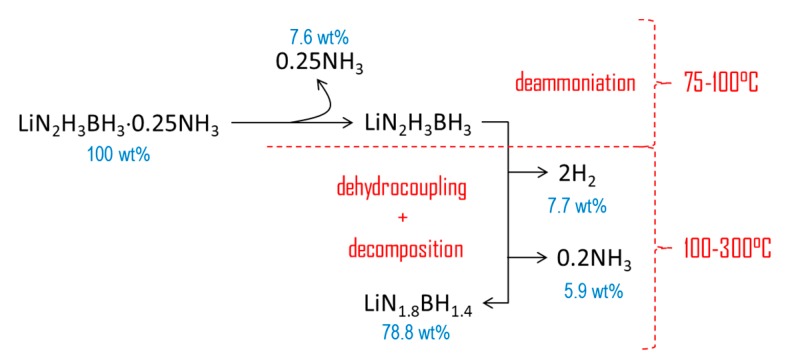
TGA results-based proposition of a decomposition mechanism of LiN_2_H_3_BH_3_·0.25NH_3_.

The new compound LiN_2_H_3_BH_3_·0.25NH_3_ was primarily synthesized for assessing its potential as chemical H storage material. Ammoniates of B–N–H compounds are of interest as, compared to their ammonia-free counterparts, they should have better dehydrogenation properties owing to the active participation of NH_3_ in the dehydrogenation process [13]. In our conditions, LiN_2_H_3_BH_3_·*x*NH_3_ has been shown to release H_2_ at temperatures lower than LiN_2_H_3_BH_3_, which is consistent with the previous remark. However, LiN_2_H_3_BH_3_·0.25NH_3_ liberates non-negligible amounts of NH_3_. According to Chua et al. [13], contamination of H_2_ by small amounts of NH_3_ is unavoidable, but in our conditions the amount of NH_3_ represents 7.6 wt% of the starting material. Under heating, the *x* molecules of NH_3_ are first released at temperatures lower than 100°C, and then the remaining solid, supposed to be mainly LiN_2_H_3_BH_3_, decomposes into H_2_ and NH_3_. Taking into account the overall weight loss of 21.2 wt% between 75 and 300 °C and the contents of H (12.1 wt%) and NH_3_ (7.6 wt%) in LiN_2_H_3_BH_3_·0.25NH_3_, there is a difference of 1.5 wt% (assuming the loss of all H atoms). This is indicative of the decomposition of LiN_2_H_3_BH_3_·0.25NH_3_ to some extent. For example, if one assumes the formation of 2 equivalents of H_2_ up to 300 °C (i.e., 7.7 wt%, which means the loss of 4 H atoms over a maximum of 6 in LiN_2_H_3_BH_3_), the proportion of NH_3_ stemming from the decomposition of LiN_2_H_3_BH_3_ would be 5.9 wt% at 300 °C. This is illustrated in Figure 6 where a mechanism of decomposition is suggested on the basis of both the present results and the results reported for LiN_2_H_3_BH_3_ elsewhere [8]. A polymeric product of unknown composition forms, similar to the residues recovered upon the dehydrogenation of most of the B–N–H compounds [5,6,7,8,9,10,11,12,27]. The nature of such residues is unknown yet because of their difficult characterization (amorphous to X-ray and too complex composition for FTIR and NMR spectroscopy techniques).

With respect to the aforementioned release of NH_3_, such a feature is unfortunately quite detrimental for the targeted application, namely chemical H storage. Indeed, the stored hydrogen is intended to be generated on demand for fueling a low-temperature fuel cell, but traces of ammonia (as low as 1 ppm) are able to severely degrade the fuel cell performance to impractical levels [28]. Given that the release of the 0.25 NH_3_ cannot be avoided with LiN_2_H_3_BH_3_·0.25NH_3_, its ammonia-free counterpart LiN_2_H_3_BH_3_ [8] seems to be more suitable for chemical H storage.

Yet, our new compound LiN_2_H_3_BH_3_·0.25NH_3_ might have potential for another application. It might be seen as a potential NH_3_ carrier as it is theoretically able to release 7.6 wt% of NH_3_ from 75 °C.

## 4. Materials and Methods

Hydrogen-storage grade LiNH_2_ was purchased from Sigma-Aldrich and used as received. Hydrazine borane N_2_H_4_BH_3_ was synthesized by salt metathesis according to an optimized procedure reported in details elsewhere [5]. Typically, an equimolar mixture of sodium borohydride NaBH_4_ (Acros Organics, Geel, Belgium) and hydrazine hemisulfate N_2_H_4_·1/2H_2_SO_4_ (Sigma-Aldrich, St Quentin Fallavier, France) in dioxane were prepared in a 250-mL three-necked round-bottom flask kept under argon flow. The mixture was allowed to react at 40 °C for 48 h. Then, the solution of N_2_H_4_BH_3_ was separated from insoluble Na_2_SO_4_ by filtration, the solvent was removed by extraction under vacuum at room temperature for 4 h, and the as-obtained borane was dried under dynamic vacuum at room temperature for 24 h. Both LiNH_2_ and N_2_H_4_BH_3_ were stored and handled in an argon-filled glove box (MBraun M200B, O_2_ < 0.1 ppm, H_2_O < 0.1 ppm).

The synthesis of our new compound was done as follows. In the argon-filled glove box, LiNH_2_ and N_2_H_4_BH_3_ were separately weighted (total of about 350 mg; equimolar ratio) and transferred in a 50-mL stainless steel jar. Several stainless steel balls (Ø 10 mm) of a total weight of 40 g were added and the jar was sealed to be taken out of the glove box. The mixture was ball-milled at ambient conditions by using a RETSCH PM 100 planetary ball mill: 2 min of milling at 300 rpm + 2 min of break, 10 times. In doing so, a paste-like solid was obtained. It was subjected to vacuum overnight. The paste-like state was modified, appearing less pasty. The sample was finally transferred into a vial to be kept in the argon-filled glove box. Note that these conditions were optimized in a preliminary systematic study (not reported herein). We indeed worked with different N_2_H_4_BH_3_/LiNH_2_ ratios (1:0.75 and 1:0.5), using different ball-milling conditions and a wet synthesis approach (solubilization/dispersion of the reactants in moisture-free organic solvents like tetrahydrofuran and dioxane). For consistency and clarity, only the optimized conditions are presented.

The molecular structure was analyzed by Fourier transform infrared (FTIR) spectroscopy (Nicolet 710, range 3600–600 cm^−1^, 32 scans). The attenuated total reflection (ATR) mode (enabling sample to be examined directly) was used at ambient conditions and under air. To minimize the contact of the sample with atmospheric O_2_ and H_2_O, a vial containing a few milligrams of the borane was prepared in the glove box, taken out, and opened just before the measurement. The number of scans was fixed to 32 to avoid excessive contact with air. In such conditions, reliable spectra are generally collected.

The molecular structure was also analyzed by ^11^B magic-angle spinning nuclear magnetic resonance (MAS NMR) spectroscopy (Varian VNMR400, 128.37 MHz, 20000 rpm, −10 °C). In the glove box, a few milligrams (or grains) of the sample was transferred into the NMR tube (Ø 10 mm) and dissolved by acetonitrile-d3 (Eurisotop, Gif sur Yvette, France). The sealed tube was then taken out of the glove box to be analyzed by NMR according to the standard procedures for this technique.

The crystal structure was analyzed by powder X-ray diffraction (PXRD) using a PANalytical X’PERT Pro multipurpose diffractometer (Cu-K_α1/α2_ radiation, λ = 1.5418 Å, 45 kV, 30 mA). All the patterns were collected using Bragg–Brentano geometry on a spinning sample holder loaded into a glove box (Jacomex PBOX, H_2_O < 5 ppm, O_2_ < 5 ppm). The powders were protected from air and oxygen contamination using a Kapton foil.

The thermal behavior of the samples was analyzed by thermogravimetric analysis (TGA; Q500 TA Instruments; heating rate 5 °C min^−1^; N_2_ flow rate 50 mL min^−1^) and differential scanning calorimetry (DSC; 2920 MDSC TA Instruments, manufacturer, New Castle, DE, USA). For both techniques, our standard conditions were as follows: sample weight of 2–3 mg; aluminum crucible of 100 mL with a pinhole (Ø 670 mm); temperature range 25–300 °C; heating rate 5 °C min^−1^; N_2_ flow rate of 50 mL min^−1^. A micro-gas chromatograph-mass spectrometer detector (µGC-MSD; S.R.A. Instruments, Agilent Technologies, Lyon, France) was used in coupling with a TGA/DSC 2 from Mettler-Toledo for the identification and quantification of the gaseous by-products. The µGC is equipped with micro-thermal conductivity detectors and two columns: one molecular sieve column for the detection and quantification of H_2_ (10 m × 0.32 mm; 5 Å; carrier gas Ar; 70 °C; head column pressure fixed at 28 psi), and one poraPLOT U column for NH_3_ (8 m × 0.15 mm i.d.; carrier gas He; 130°C; head column pressure fixed at 30 psi).

## Figures and Tables

**Table 1:** Space group (s.g.), unit cell parameters, goodness of fit, and R-values for the refined structures for **3** (LiN_2_H_3_BH_3_·*x*NH_3_) and **2** (LiNH_2_) at room temperature.

	LiN_2_H_3_BH_3_·*x*NH_3_	LiNH_2_
s.g.	*P2*_1_/*n* (N° 14)	$I\bar{4}$ (N° 82)
Z	4	8
a (Å)	7.6498(18)	5.1158(11)
b (Å)	7.482(3)	5.1158(11)
c (Å)	5.968(17)	10.103(3)
β (°)	97.803(12)	-
V (Å^3^)	338.91(17)	264.41(12)
R.P.A. (wt%) ^1^	95.4(5)%	4.6(6)%
GoF	2.94	2.94
Rp	3.66	3.66
wRp	486	4.86
R(obs)/R(all)	13.85/15.47	11.17/12.49
wR(obs)/wR(all)	11.66/11.77	11.92/12.00

^1^ Relative phase amounts in weight.

**Table 2:** Experimental structural parameters of **3** (LiN_2_H_3_BH_3_·*x*NH_3_) and **2** (LiNH_2_) at room temperature. The atomic positions for LiNH_2_ were kept fixed during the refinement [19].

Sample	Atom	Site	Occupancy	x	y	z	U_iso_ (Å^2^)
**3**	Li1_1	4e	1	0.4025(11)	0.4401(9)	0.767(2)	0.0229(1)
	B2_1	4e	1	0.7210(11)	0.3147(17)	0.5489(13)	0.0213(1)
	N3_1	4e	1	0.6451(11)	0.2980(11)	0.7827(11)	0.0202(1)
	N4_1	4e	1	0.6373(18)	0.1096(14)	0.867(3)	0.0202(1)
**2**	Li1_2	2a	1	0.00000	0.500000	0.25000	0.0177(1)
	Li2_2	2d	1	0.00000	0.00000	0.00000	0.0177(1)
	Li3_2	4e	1	0,00000	0.00000	0.25300	0.0177(1)
	N4_2	8g	1	0.23400	0.25400	0.13700	0.0065(1)
